# Assessment of Conventional Oxygen Therapy, High-Flow Nasal Cannula, and Non-Invasive Ventilation to Secure Bronchofiberoscopy in Patients with Respiratory Acidosis: A Narrative Review and a Proposal for a Protocol in View of a Randomized Multicenter Study

**DOI:** 10.3390/jcm15103960

**Published:** 2026-05-21

**Authors:** Mikołaj Rycerski, Adam Warcholiński, Michał Zieliński, Federico Longhini, Mrinal Sircar, Aleksandra Oraczewska, Magdalena Latos, Patrycja Rzepka-Wrona, Szymon Białka, Grzegorz Brożek, Szymon Skoczyński

**Affiliations:** 1 Department of Lung Diseases and Tuberculosis, Faculty of Medical Sciences in Zabrze, Medical University of Silesia in Katowice, 41-803 Zabrze, Poland; michal.zielinski@sum.edu.pl (M.Z.); oraczewskaaleksandra@gmail.com (A.O.); magdalena.latos@sum.edu.pl (M.L.); przepka-wrona@sum.edu.pl (P.R.-W.); sz.skoczynski@sum.edu.pl (S.S.); 2 ATEMIDA Sp. Z o.o., 40-875 Katowice, Poland; adam.warcholinski@gmail.com; 3 Department of Medical and Surgical Sciences, Anesthesia and Intensive Care, Magna Graecia University of Catanzaro, 88100 Catanzaro, Italy; longhini.federico@gmail.com; 4 Pulmonology and Critical Care Fortis Hospital, NOIDA Sector 62, Noida 201301, India; mrinalsircar@yahoo.co.uk; 5 Department of Anaesthesiology and Intensive Care, Faculty of Medical Sciences in Zabrze, Medical University of Silesia in Katowice, 41-803 Zabrze, Poland; szymon.bialka@gmail.com; 6 Department of Epidemiology, Faculty of Medical Sciences in Katowice, Medical University of Silesia, 40-055 Katowice, Poland; gbrozek@sum.edu.pl

**Keywords:** fiberoptic bronchoscopy (FOB), high-flow nasal cannula (HFNC), non-invasive ventilation (NIV), conventional oxygen therapy (COT), lung disease diagnosis, chronic respiratory acidosis, type 2 respiratory failure

## Abstract

**Background**: Fiberoptic bronchoscopy (FOB) is a procedure routinely performed in clinical practice for both diagnostic and therapeutic purposes. FOB frequently impairs respiratory function, which may exacerbate respiratory failure. Currently, conventional oxygen therapy (COT) is the most commonly used form of respiratory support; however, non-invasive ventilation (NIV) and high-flow nasal cannula (HFNC) are being used increasingly. The optimal settings and indications for NIV and HFNC in patients with respiratory acidosis undergoing FOB have not yet been determined. **Methods**: This is a prospective, multicenter, randomized controlled trial including two parallel study populations defined by the indication for bronchoscopy and the type of respiratory acidosis. Therapeutic FOB (Study 1): Patients with decompensated type 2 respiratory failure (pH < 7.35 and PaCO_2_ > 45 mmHg) will be randomized to receive one of four methods of respiratory support during bronchoscopy: COT, NIV, HFNC, or invasive mechanical ventilation (IMV) (*n* = 315). Diagnostic FOB (Study 2): Patients with chronic respiratory acidosis (pH ≥ 7.35, PaCO_2_ > 45 mmHg, and/or HCO_3_^−^ > 27 mmol/L) will be randomized to receive COT, NIV, or HFNC during bronchoscopy (*n* = 210). Before FOB, patients in both groups will undergo arterial blood gas (ABG) analysis. During FOB, vital signs will be continuously monitored, including SpO_2_, FiO_2_, TcCO_2_, ECG, and heart rate. After FOB, ABG analysis will be repeated, and study endpoints and complications, if any, will be recorded. The planned study period is from April 2026 to April 2029. **Results**: Based on the study results, we aim to evaluate the effectiveness and safety of different respiratory support strategies during flexible bronchoscopy, with the primary objective of comparing the rate of treatment failure among COT, HFNC, NIV, and IMV. Treatment failure is defined as the need for endotracheal intubation, premature termination of the procedure, or escalation of respiratory support. Additionally, we aim to identify the optimal NIV and HFNC settings, as well as complication rates in both study groups. **Conclusions**: The results of this study will help define the role of optimal respiratory support in patients with respiratory acidosis undergoing FOB, potentially leading to a shorter time from admission to diagnosis, better tolerance of the procedure, and faster recovery afterward.

## 1. Introduction

Fiberoptic bronchoscopy (FOB) is a safe procedure with high diagnostic value in respiratory diseases. It is used in the evaluation of hemoptysis, interstitial lung diseases, respiratory tract neoplasms, and mediastinal lymphadenopathy. In addition, FOB can be used therapeutically, for example, in cases of foreign body aspiration and to restore airway patency in patients with pneumonia or tumors [1].

The safe performance of FOB requires appropriate monitoring, and adherence to standardized procedural protocols. According to the available literature and clinical guidelines, continuous assessment of oxygenation, hemodynamic status, and ventilation is essential, particularly in patients with impaired respiratory function. Although the use of supplemental oxygen is recommended in the majority of patients, at-risk patients may require more advanced respiratory support strategies. Furthermore, the presence of experienced personnel and the ability to promptly recognize and manage complications are considered key elements of safe bronchoscopy practice. Despite these recommendations, optimal strategies for respiratory support during bronchoscopy, especially in patients with respiratory failure, remain insufficiently defined [2,3].

FOB may impair respiratory function, resulting in reduced minute ventilation, hypoxemia, and hypercapnia. Although these effects are generally well tolerated, they may lead to clinically significant deterioration, particularly in patients with underlying respiratory failure, and may increase the risk of cardiopulmonary complications [2].

FOB carries a risk of serious complications, including decreased minute ventilation and worsening secondary hypoxemia. It may also predispose patients to hypercapnia, which can contribute to the exacerbation of respiratory failure and, secondarily, increase the risk of cardiac and pulmonary complications [3].

Sedation is commonly used during flexible bronchoscopy to improve patient comfort and tolerance of the procedure [2,4]. However, sedative medications may impair respiratory pattern to a varying extent [5,6], particularly in patients with underlying respiratory failure, leading to a secondary gas exchange impairment. Although the most common mode to guarantee patient oxygenation remains conventional oxygen therapy (COT), some other approaches have been proposed, such as high-flow nasal cannula (HFNC) and non-invasive ventilation (NIV), to counterbalance the effects of FOB and sedation on gas exchange impairment [7,8,9].

COT refers to oxygen delivery using standard low- or moderate-flow devices, such as a nasal cannula or simple face mask. During FOB, oxygen is usually delivered via nasal cannula, as face masks hamper the insertion of the bronchoscope [7,8,9]. In contrast to HFNC and NIV, conventional oxygen therapy does not actively support ventilation or reduce the work of breathing.

HFNC is an alternative technique to oxygenate patients by the delivery of high flow (up to 60 L/min) of air/oxygen admixture with an inspired oxygen fraction (FiO_2_) ranging between 21% and 100%, heated at 31–37 °C and fully humidified. HFNC has the advantages to generate a flow- and time-dependent low level of positive end-expiratory pressure, to washout the oropharyngeal dead space from CO_2_, leading to better breath efficiency and respiratory effort reduction. In addition, HFNC prevents alveola derecruitment during FOB procedures and guarantees a better oxygenation [10,11].

As opposed to COT and HFNC, NIV is a ventilatory support, rather than an oxygenation strategy alone, since it applies different inspiratory and expiratory pressure to the airway throughout the patient’s breath. NIV has also been evaluated during FOB, and it was shown to be able to reduce respiratory effort and dyspnea, while guarantying better gas exchange, as compared to COT [12,13,14]. In addition, it enables the safe performance of both diagnostic and therapeutic FOB in patients with exacerbations of respiratory failure and in those with severe chronic respiratory failure [9].

In a recent multicenter randomized trial conducted by Qin et al. in 2025 [15], HFNC was compared to COT in a group of patients with hypoxemia and/or hypercapnia before FOB. HFNC was shown to significantly reduce the rate of desaturation, procedure interruption, and need for treatment escalation. Additionally, compared to COT, both HFNC and NIV have been recently shown to reduce the hypoxemic events during FOB in chronic obstructive pulmonary disease (COPD) patients [15,16].

Recent guidelines from the European Society of Anaesthesiology and Intensive Care (ESAIC) and the European Society of Intensive Care Medicine (ESICM) suggest the use of non-invasive respiratory support methods rather than COT in hypoxemic patients undergoing FOB [17].

COPD patients, as like as other patients suffering from disorders characterized by alveolar hypoventilation such as obesity hypoventilation syndrome (OHS), neuromuscular diseases, severe chest wall deformities, or advanced bronchiectasis, develop chronic hypercapnia that is physiologically compensated by the kidney by bicarbonate (HCO_3_^−^) retention. Whenever an exacerbation of the chronic respiratory failure ensues, respiratory acidosis may develop due to further ventilation impairment and rapid increment of arterial partial pressure of carbon dioxide (pCO_2_).

Compensated respiratory hypercapnia is typically characterized by pCO_2_ > 45 mmHg with pH between 7.35 and 7.45 (normal values) and elevated HCO_3_^−^, reflecting renal compensation. In case of acute-on-chronic respiratory failure or an acute failure of the respiratory muscle pump function, respiratory acidosis develops and is characterized by arterial pH < 7.35 and increased pCO_2_ values (>45 mmHg)

Currently, there is no strong evidence to suggest that NIV-assisted FOB and/or HFNC-assisted FOB are superior in patients with chronic respiratory failure. There are also no precisely defined device settings for these patients. Detailed indications and contraindications for these procedures have not yet been established, nor have the optimal parameters of respiratory support in patients with respiratory acidosis been determined.

Due to the increasing number of critically ill patients with respiratory failure, COT is often insufficient to ensure safe FOB performance [8,18].

Intubation and invasive mechanical ventilation (IMV) also represent an important method of respiratory support during FOB. They should be considered before FOB in patients with severe hypoxemia [19]. However, this mainly applies to patients undergoing FOB for therapeutic purposes, because the standard approach is first to treat respiratory acidosis before performing diagnostic FOB. In at-risk patients who have not been intubated before the procedure, FOB may exacerbate respiratory failure, leading to the need for urgent intubation and transfer to the intensive care unit (ICU). During FOB in intubated patients, insertion of the bronchoscope through the endotracheal tube significantly reduces the effective internal diameter of the tube, thereby increasing airway resistance and potentially requiring higher airway pressures to maintain adequate ventilation. This may contribute to complications associated with positive-pressure ventilation, including barotrauma [20].

Despite existing recommendations regarding monitoring and general safety principles during bronchoscopy, there is still limited high-quality evidence defining the optimal respiratory support strategy and its settings in patients with respiratory failure, particularly in those with acute or chronic type 2 respiratory failure.

For this reason, strategies that provide adequate respiratory support during FOB without the need for intubation are of particular clinical interest. Non-invasive approaches, such as HFNC and NIV, may help maintain oxygenation and ventilation during FOB while potentially avoiding complications associated with invasive mechanical ventilation.

According to the available literature, both HFNC and NIV are superior to COT in preventing hypoxemia during FOB, as well as in reducing CO_2_ retention. Therefore, we hypothesize that both methods will be superior to COT during FOB in patients with acute and chronic type 2 respiratory failure.

To perform FOB in a manner that is safe and well tolerated in patients with both compensated and decompensated type 2 respiratory failure, it is necessary to determine the most appropriate respiratory support methods.

With this randomized controlled trial, we aim to assess the potential of NIV and HFNC, as well as their optimal application during therapeutic and diagnostic FOB in patients with both compensated and decompensated chronic respiratory failure.

## 2. Materials and Methods

### 2.1. Study Design and Population

The protocol describes a prospective, multicenter, randomized controlled trial comprising two parallel study populations with separate randomization. In the therapeutic FOB group (Study 1), patients will be randomized to one of four respiratory support strategies. In the diagnostic FOB group (Study 2), patients will be randomized to one of three respiratory support strategies.

The study protocol was developed in accordance with the Declaration of Helsinki and its subsequent amendments [21]. Both protocols were approved by the Bioethics Committee of the Medical University of Silesia (SUM BC), Resolution Nos. BNW/NWN/0052/KB1/96/I/24/25 and BNW/NWN/0052/KB1/95/I/24/25. However, each participating center will be required to obtain approval from its local bioethics committee before enrolling patients in the study.

The study will include adult patients (women and men) who provide informed consent to participate. Recruitment will be conducted among patients aged 18 years or older with indications for either therapeutic or diagnostic FOB, with no upper age limit.

Eligibility assessment will be conducted under conditions ensuring privacy and compliance with the General Data Protection Regulation (GDPR) [22]. Patients will be thoroughly informed about the course of the study, encouraged to ask questions, and provided with full clarification of any concerns. They will then be asked to provide written informed consent to participate in the research project.

Patients will be assigned to one of two studies according to the indication for bronchoscopy and their respiratory parameters: therapeutic FOB (Study 1) or diagnostic FOB (Study 2). Patients undergoing therapeutic FOB (Study 1) will be in a more severe clinical condition; therefore, patients with exacerbated chronic respiratory acidosis and respiratory failure (pCO_2_ > 45 mmHg and pH < 7.35 on arterial blood gas analysis performed before FOB) will be recruited. In the diagnostic arm (Study 2), only patients with chronic respiratory failure (pCO_2_ > 45 mmHg and/or HCO_3_^−^ > 27 mmol/L, but pH ≥ 7.35) will be recruited.

Patients in the therapeutic FOB group (Study 1) will be divided into three subgroups according to the severity of acidosis demonstrated on ABG analysis: pH < 7.2, 7.2 ≤ pH < 7.3, and 7.3 ≤ pH < 7.35. Randomization to the respiratory support method will be performed within these strata. Patients are stratified into three pH categories to reflect clinically relevant gradations in the severity of hypercapnic respiratory failure. This approach allows a more detailed assessment of treatment effects across different levels of respiratory compromise.

These criteria are commonly used in clinical decision-making regarding escalation of respiratory support, particularly when distinguishing patients who require non-invasive ventilation from those who require invasive mechanical ventilation [23,24].

The planned total sample size is 525 patients, including 315 patients undergoing FOB for therapeutic purposes and 210 patients undergoing FOB for diagnostic purposes.

The target sample size of 525 patients across all groups was calculated based on the risk of complications in the form of intubation during FOB in patients with respiratory failure, with a frequency estimated at 0.2–2% [25,26]. This includes 315 patients undergoing FOB for therapeutic reasons and 210 patients undergoing FOB for diagnostic reasons (Table 1 and Table 2). Based on the level of interest among centers willing to cooperate in the study, it may be possible to recruit a larger number of patients, allowing for a more comprehensive analysis.

In the subgroup of the most severely ill patients in the therapeutic FOB group (pH < 7.2), interim analyses will be performed after the recruitment of 15, 30, and 45 patients, respectively, in order to monitor study safety in the group at the highest risk.

### 2.2. Inclusion and Exclusion Criteria

#### 2.2.1. Inclusion Criteria

In Study 1, we will include adult (e.g., ≥18 years/old) patients with the indication of FOB for therapeutic purposes (i.e., pneumonia in patients with an impaired cough reflex; bronchial toilet and microbiological sample collection; management of respiratory tract hemorrhage using fiberoptic bronchoscopy; removal of foreign bodies; and relief of bronchial obstruction in advanced neoplastic disease) and arterial blood gases showing a pCO_2_ > 45 mmHg and pH < 7.35.

In Study 2, we will include only adult (e.g., ≥18 years/old) patients with the indication of FOB for diagnostic purpose (i.e., suspected lung cancer, suspected sarcoidosis, mediastinal lymphadenopathy) and arterial blood gases showing a pH ≥ 7.35 with pCO_2_ > 45 mmHg and/or HCO_3_^−^ > 27 mmol/L.

In both study groups, written informed consent to participate in the study will be mandatory.

#### 2.2.2. Exclusion Criteria

Patients in Study 1 will be excluded if showing one or more of the following criteria:

- Unstable coronary artery disease chronic coronary syndrome (CCS) III/IV, circulatory failure New York Heart Association (NYHA) III/IV (does not apply to tests for vital indications, e.g., aspiration of a foreign body);
- Hemodynamic instability defined as mean arterial pressure (MAP) < 65 mmHg despite fluid resuscitation or the requirement for vasopressor support, constant use of pressor amines, myocardial infarction in the last 2 weeks without percutaneous coronary intervention (PCI) treatment, unstable angina pectoris, or severe arrhythmias (e.g., sustained ventricular tachycardia, ventricular fibrillation, unstable supraventricular tachyarrhythmias);
- Chronic primary pulmonary hypertension, assessed during right heart catheterization World Health Organization Functional Class (WHO) III/IV;
- Pneumothorax not secured with drainage;
- Platelet count < 20,000/µL, if platelets are not transfused immediately before or during the procedure;
- International normalized ratio (INR) > 2 or activated partial thromboplastin time (APTT) > 45 s. [27];
- Anemia: hemoglobin (Hb) level < 8 g/dL or 8–10 g/dL if the physician prescribes a blood transfusion;
- Patients who were intubated before randomization.

In Study 2, patients will be excluded if showing one or more of the following criteria:

- Coronary artery disease CCS III/IV;
- Hemodynamic instability defined as mean arterial pressure (MAP) < 65 mmHg despite fluid resuscitation or the requirement for vasopressor support, recent myocardial infarction within the last 2 weeks (regardless of PCI), unstable angina, or severe arrhythmias (e.g., sustained ventricular tachycardia, ventricular fibrillation, unstable supraventricular tachyarrhythmias). Pneumothorax not secured with drainage;
- Platelet count < 20,000/µL, if platelets are not transfused immediately before/during the procedure;
- INR > 1.5 or APTT > 40 s [27];
- Hemoglobin (Hb) level < 10 g/dL;
- Patients who were intubated before randomization.

### 2.3. Data Collection

#### 2.3.1. Before FOB

**Figure 1:**
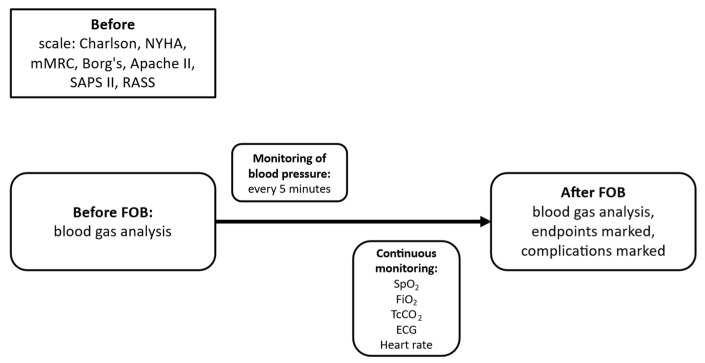
Performing FOB for therapeutic reasons, data collection.

**Figure 2:**
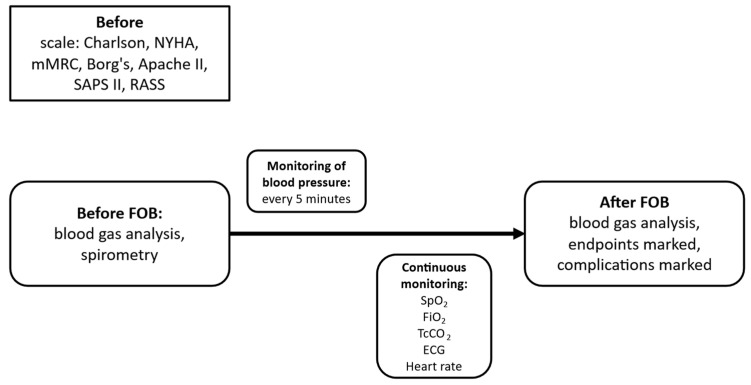
Performing FOB for diagnostic reasons, data collection.

**Figure 3:**
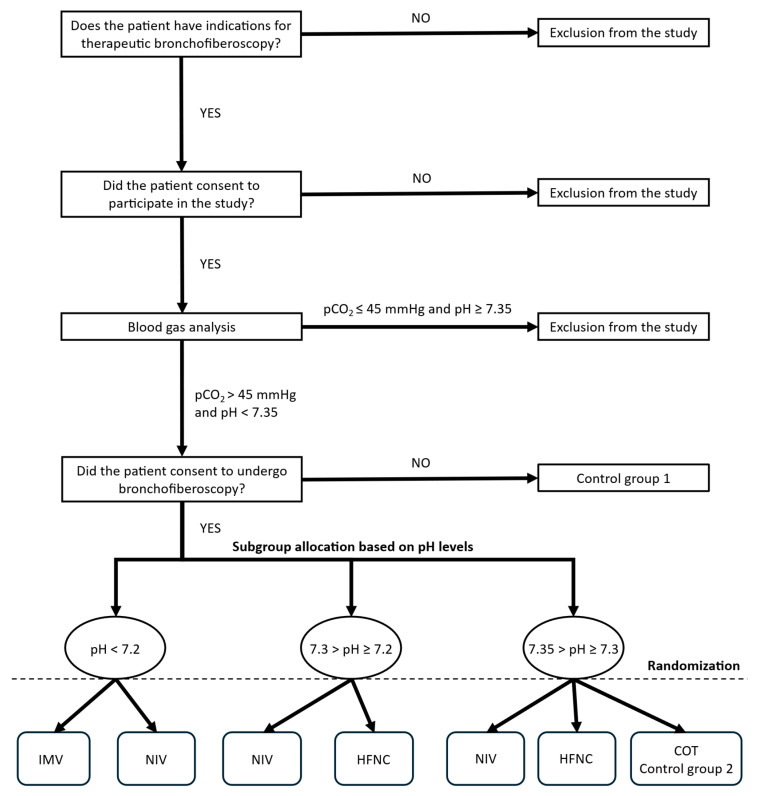
Randomization of patients undergoing FOB for therapeutic reasons (Study 1).

**Figure 4:**
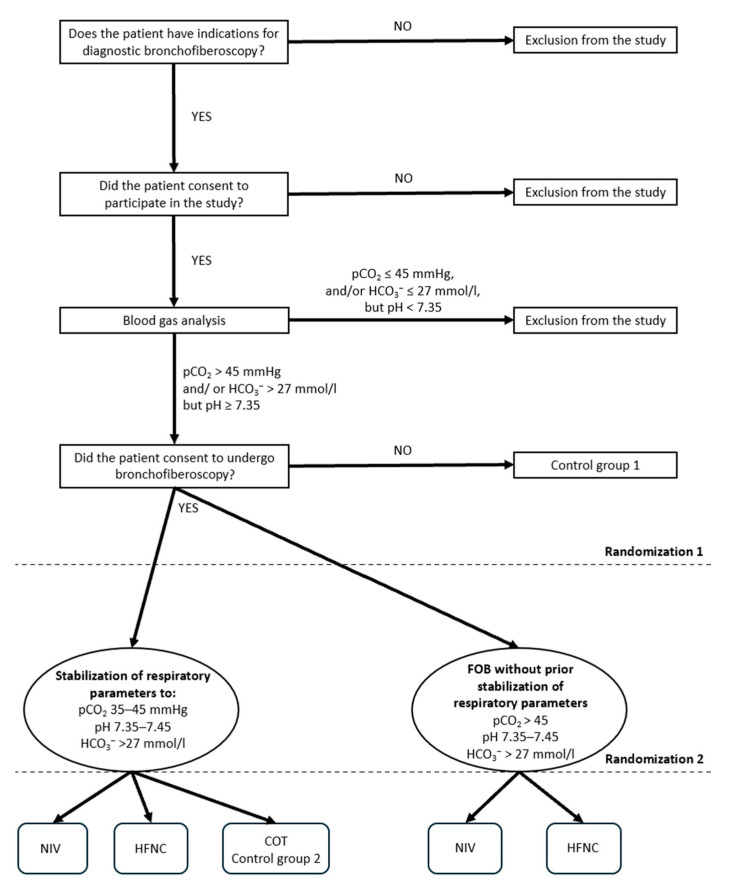
Randomization of patients undergoing FOB for diagnostic reasons (Study 2).

A detailed medical history will be obtained from all patients, including comorbidities, current medications, smoking history, and the results of the following assessments: New York Heart Association (NYHA) classification, modified Medical Research Council (mMRC) dyspnea scale, Charlson Comorbidity Index, Borg scale, Acute Physiology and Chronic Health Evaluation II (APACHE II), Simplified Acute Physiology Score II (SAPS II), and Richmond Agitation-Sedation Scale (RASS). In patients undergoing diagnostic FOB, pulmonary function testing (spirometry) will be performed if the patient’s clinical condition permits. Blood pressure, oxygen saturation, and arterial blood gases (approximately 2.0 mL) will be measured. Arterial blood gas analysis will be performed both before and after the procedure (Figure 1 and Figure 2). After qualification to a specific study group (Figure 3 and Figure 4), patients will be randomly assigned to a respiratory support method.

Before FOB, intravenous sedative medications will be administered by the bronchoscopist, most likely as a combination of fentanyl and midazolam.

Midazolam and fentanyl will be administered in accordance with the British Thoracic Society (BTS) guidelines, with the aim of achieving a sedation level of −2 to −3 on the Richmond Agitation-Sedation Scale (RASS), corresponding to 3 to 4 on the Ramsay scale. Sedation is typically achieved using midazolam in combination with an opioid, such as fentanyl. The depth of sedation will be continuously monitored, and sedative agents will be titrated accordingly [2].

Midazolam will be administered as a slow intravenous injection at a maximum rate of 2 mg/min. An initial dose of 2.5 mg will be given 5 to 10 min before the procedure. If necessary, supplementary doses of 1 mg will be administered 2 to 10 min after the previous dose. In elderly patients (≥70 years), patients with low body weight, and patients with chronic kidney disease and/or heart failure, the initial dose will be reduced to 1 mg.

Fentanyl will be administered as a slow intravenous injection, with an initial dose of 25 µg. If necessary, supplementary doses of 25 µg will be given 3 to 5 min after the previous dose.

When both fentanyl and midazolam are used, fentanyl will be administered before midazolam.

The study protocol does not mandate a specific sedative regimen, to respect local guidelines and local practice, including the possible use of propofol-based sedation depending on operator preference.

Topical airway anesthesia, such as lidocaine, will be used according to local practice to improve procedural tolerance and reduce the cough reflex. An initial dose of 10% lidocaine spray, administered as three actuations (30 mg), will be given before the procedure.

#### 2.3.2. Monitoring During FOB

During the entire procedure, the following parameters will be monitored: SpO_2_, FiO_2_, TcCO_2_, ECG, and heart rate, all of which will be recorded continuously, as well as non-invasive arterial blood pressure, which will be measured every 5 min (Figure 1 and Figure 2). Transcutaneous CO_2_ monitoring will be performed using calibrated devices in accordance with the manufacturer’s recommendations. Although TcCO_2_ provides a continuous, non-invasive estimate of PaCO_2_ trends, arterial blood gas analysis remains the reference standard and will be performed in cases of clinical deterioration.

Particular attention will be paid to the potential risk of airway obstruction during FOB, especially in the context of ventilation interfaces. The presence of the bronchoscope within the airway may increase airflow resistance and reduce the effective internal diameter of the airway or endotracheal tube, particularly in patients receiving NIV or IMV, and this will be taken into account during ventilatory management.

#### 2.3.3. After FOB

After FOB, arterial blood gas analysis will be repeated, and study endpoints and complications will be recorded. These complications will include bronchospasm, hypoxemia, decompensated respiratory acidosis, local bleeding, fever, the need to interrupt the procedure, transfer to the ICU, pneumothorax, and death [28,29].

FOB will be performed using optical or video bronchofiberscopes or endobronchial ultrasound (EBUS) devices. The diameter of the bronchofiberscope used will be recorded, as well as the type and dose of sedation administered during FOB in each randomized group, including the initial, supplementary, and total doses. The target depth of sedation will be a RASS score between −2 and −3, although any deviation from this target range will also be documented. In addition, sedation-related adverse events will be recorded, including the use of antidotes to sedative agents (e.g., flumazenil and naloxone) and the doses administered.

Data obtained from the interview and additional tests will be entered anonymously into a computer database in compliance with GDPR requirements and will then be statistically analyzed. Appropriate conclusions will be drawn based on the results obtained.

### 2.4. Randomization

Randomization will be performed using a centralized, computer-generated sequence via a dedicated online platform. Allocation concealment will be ensured by the system, with treatment assignment revealed only after patient enrollment.

Due to the nature of the interventions, blinding is not feasible; however, outcomes will be assessed using objective, predefined criteria.

In the group undergoing FOB for therapeutic reasons, patients will be randomized after assignment to one of the previously described subgroups based on arterial pH. Randomization will then be stratified according to the predefined pH-based subgroups with respect to the specific respiratory support method (Study 1).

In patients undergoing diagnostic FOB, a two-step randomization process will be used. First, patients will be randomized into two categories to determine whether stabilization of respiratory parameters should be performed before the procedure or not (Study 2).

Therapeutic FOB (Study 1), randomization within shown groups (Figure 3 and Table 1):

1. pH < 7.2
  - IMV
  - NIV
2. 7.2 ≤ pH < 7.3
  - NIV
  - HFNC
3. 7.3 ≤ pH < 7.35
  - NIV
  - HFNC
  - Conventional oxygen therapy

For diagnostic FOB (Study 2), two-step randomization (1st randomization: 1 or 2; 2nd randomization: a–c or a–b method of respiratory support) (Figure 4 and Table 2):

1. Stabilization of respiratory parameters before FOB (pH 7.35–7.45; pCO_2_ 35–45 mmHg; HCO_3_^−^ >27 mmol/L)
  - NIV
  - HFNC
  - Conventional oxygen therapy
2. FOB right away (pH 7.35–7.45; pCO_2_ > 45 mmHg; HCO_3_^−^ > 27 mmol/L)
  - NIV
  - HFNC

### 2.5. Course of the Study and Escalation of Therapy

When randomized to the HFNC group, the patient will be fitted with dedicated large-bore nasal cannulas specifically designed, after which FOB will be performed. The use of nasal cannulas does not interfere with bronchoscope insertion, which is performed orally.

**Figure 5:**
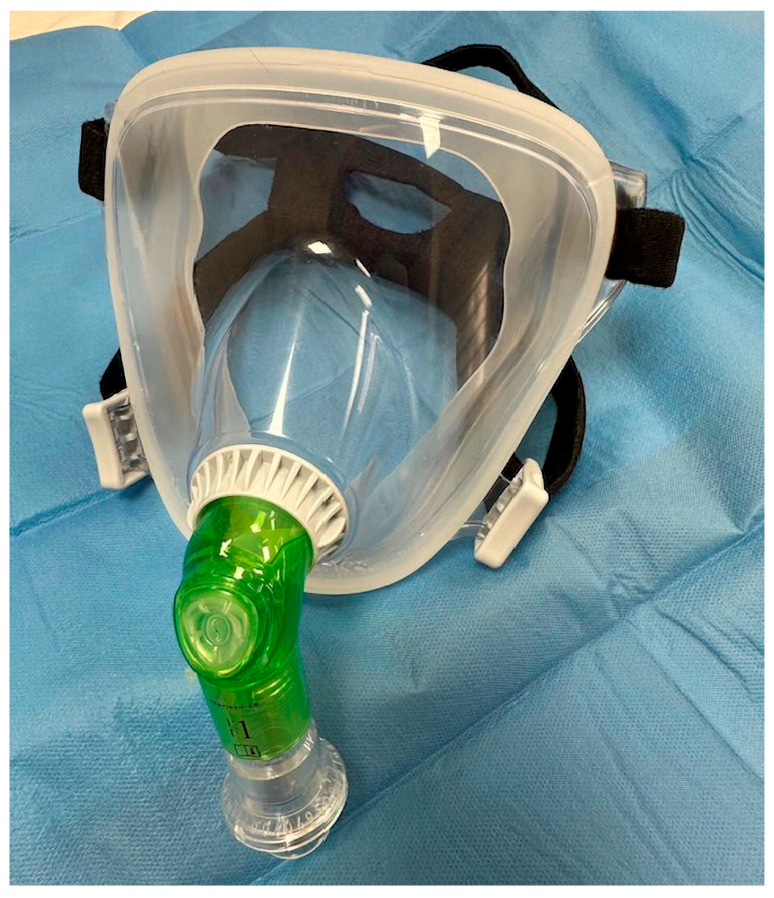
NIV full-face mask with bronchoscopic elbow.

In the NIV group, ventilation will be delivered through a full-face mask connected to the ventilator via a dedicated bronchoscopic elbow adapter positioned between the mask and the ventilator circuit. The adapter includes a sealed bronchoscopy port with a silicone membrane that allows insertion of the bronchoscope while maintaining circuit pressure and minimizing air leakage. This configuration enables bronchoscopy to be performed without interrupting non-invasive ventilation (Figure 5).

Throughout the procedure, a predefined safety protocol will be applied.

The study will compare the initial settings of the devices, which will be maintained provided that the examination is carried out correctly and the patient’s clinical condition is stable.

#### 2.5.1. Escalation of Therapy

Escalation of therapy is defined according to the principle of progression to a higher level of respiratory support (e.g., COT → HFNC → NIV → IMV) in response to clinical deterioration, rather than for technical or procedural reasons. No minimum duration of treatment with a given modality is required before escalation. Escalation must be clinically justified and sustained, rather than limited to transient adjustments.

The initial settings of the assigned device may be modified according to the principle of titration if a trend toward desaturation or hypercapnia is observed. Desaturation is defined as a decrease in SpO_2_ of 1% to <10% from the preprocedural value, sustained for at least 60 s. Hypercapnia is defined as an increase in TcCO_2_ of 1 to <10 mmHg from the preprocedural value, sustained for at least 60 s.

Indications for escalation of respiratory support include desaturation, defined as a decrease in SpO_2_ of ≥10% from the preprocedural value sustained for at least 60 s; hypercapnia, defined as an increase in TcCO_2_ of ≥10 mmHg from the preprocedural value sustained for at least 60 s; or other abnormalities identified during clinical assessment of the patient. If any of the above occur, immediate escalation of therapy is indicated.

#### 2.5.2. Termination of FOB

If these abnormalities persist despite escalation of respiratory support (i.e., a decrease in SpO_2_ of ≥10% for at least 60 s and/or an increase in TcCO_2_ of ≥10 mmHg for at least 60 s), arterial blood gas analysis may be performed during the procedure. If arterial blood gas analysis confirms worsening respiratory acidosis compared with the preprocedural ABG, particularly a further decrease in pH and/or an increase in PaCO_2_ of more than 10 mmHg relative to the initial value, the examination will be interrupted. The procedure will also be terminated if clinical deterioration continues and blood gas analysis confirms worsening gas exchange or respiratory acidosis.

#### 2.5.3. Intubation

In the study concerning therapeutic FOB (Study 1), intubation is included as part of the randomization for patients in the pH < 7.2 group and may also serve as a possible method of escalation in patients randomized to NIV within this subgroup. However, patients in the same study who are assigned to the 7.2 ≤ pH < 7.3 group (randomized to HFNC or NIV) or to the 7.3 ≤ pH < 7.35 group (randomized to COT, HFNC, or NIV) may also deteriorate. Therefore, to ensure safe performance of therapeutic FOB, escalation to IMV or termination of the procedure will be permitted when clinically indicated.

In patients with acute indications for therapeutic FOB, continuation of the procedure under IMV may be clinically justified. In such scenarios, intubation may allow safe completion of the procedure and may provide greater clinical benefit than premature termination, particularly when the underlying condition requires immediate intervention.

Patients undergoing diagnostic FOB (Study 2), who are randomized to COT, HFNC, or NIV, are less likely to deteriorate to the point of requiring intubation, although this remains a possible outcome, as it is one of the recognized complications of FOB [25,27]. In this group, in the event of clinical deterioration, the preferred approach is early termination of the procedure rather than escalation to invasive mechanical ventilation, as the diagnostic nature of the intervention does not usually justify continuation under increased procedural risk.

Final decisions regarding escalation of respiratory support, early termination of the procedure, or intubation will remain at the discretion of the bronchoscopist and must be documented after the procedure.

### 2.6. Device Settings

The following device settings are based on commonly used clinical ranges and are intended to standardize respiratory support across study groups while preserving the flexibility required for individualized adjustment according to each patient’s clinical condition. In all study arms, the bronchoscope will be inserted orally.

For HFNC, a high flow rate will be used to ensure consistent physiological effects. Although HFNC may generate a low level of positive airway pressure, this effect depends on factors such as whether the patient breathes through the mouth or nose, and is not considered the primary therapeutic mechanism. The selected flow rate of 60 L/min reflects common clinical practice and is intended to provide adequate CO_2_ washout and stable oxygen delivery during the procedure. Because partial upper airway obstruction by the bronchoscope may reduce the effective flow delivered during bronchoscopy, clinical response and gas exchange will be closely monitored throughout the examination.

For NIV, the values provided represent initial settings and upper titration limits rather than fixed therapeutic targets, reflecting the need for individualized adjustment based on respiratory mechanics, gas exchange, and patient tolerance. In patients with COPD or suspected intrinsic PEEP, EPAP will be titrated to partially offset auto-PEEP, with the aim of reducing inspiratory effort while avoiding excessive dynamic hyperinflation. NIV will be initiated at lower pressure support levels and subsequently adjusted according to clinical response, gas exchange, and tolerance.

Across all respiratory support modalities, parameters will be titrated to maintain adequate oxygenation and ventilation while ensuring patient safety. Escalation to more advanced respiratory support, including invasive mechanical ventilation, will be permitted at any stage of the procedure when clinically indicated.

All device settings, as well as any adjustments or changes made during the procedure, will be documented to ensure consistency and enable comparison across participating centers. This documentation will include the need for escalation of respiratory support and any deviation from the assigned respiratory support method.

1. For therapeutic FOB (Study 1):
  - HFNC, flow 60 L/min, temperature 34 °C and FiO_2_ under saturation control to achieve SpO_2_ ≥ 92%. Baseline FiO_2_—equivalent to FiO_2_ during oxygen therapy before FOB (during hospitalization).
    Humidification will be provided according to device-specific settings and local clinical practice, as available HFNC systems differ in their ability to adjust humidity parameters.
  - NIV will be conducted in ST mode (spontaneous to forced), number of breaths 16–18/min, EPAP (positive expiratory pressure) 6–14 cm H_2_O, Tins (duration) 0.8–1 s, PS (pressure support ventilation) 8–25 cm H_2_O and oxygen flow under saturation control to achieve SpO_2_ ≥ 92%. Baseline oxygen with FiO_2_—at the level of previously administered FiO_2_, trigger and low cycle.
  - IMV: device parameters will correspond to previous settings to achieve SpO_2_ ≥ 92%, based on minute ventilation and tidal volume. The initial mode used should be recorded, including PS, EPAP, and FiO_2_. For intubated patients, the endotracheal tube size should be recorded.
2. For diagnostic (Study 2):
  - HFNC, flow 60 L/min, temperature 34 °C and FiO_2_ under saturation control to achieve SpO_2_ ≥ 92%.
    Humidification will be provided according to device-specific settings and local clinical practice, as available HFNC systems differ in their ability to adjust humidity parameters.
  - NIV will be conducted in ST mode (spontaneous to forced), number of breaths 16–18/min, EPAP (expiratory positive pressure) 6–12 cm H_2_O, Tins (duration) 0.8–1.1 s, PS 12–23 cm H_2_O and oxygen flow under saturation control to achieve SpO_2_ ≥ 92%.

### 2.7. Statistical Analysis

The target planned number of 525 patients studied from all groups was calculated based on the risk of complications in the form of intubation during FOB in patients with respiratory failure with a frequency estimated at 0.2–2% [25,26]. In that number, we account for 315 patients undergoing FOB for therapeutic reasons, and 210 undergoing FOB for diagnostic reasons.

Assuming a normal distribution, a significance level of 0.05, and a statistical power of 0.95, the sample size was calculated using a medium standardized effect size (Cohen’s d = 0.5). This value was chosen due to its representation of a clinically meaningful difference between respiratory support strategies that could influence the safety of FOB in patients with acute and chronic respiratory acidosis while remaining realistic based on previously reported physiological and clinical effects of HFNC and NIV during bronchoscopy.

Statistical analysis will be performed using standard methods depending on data distribution. Continuous variables will be tested for normality using the Shapiro–Wilk test and presented as mean ± standard deviation or median (IQR), as appropriate. Comparisons between groups will be performed using one-way ANOVA or the Kruskal–Wallis test for continuous variables, and Chi-squared or Fisher’s exact test for categorical variables.

Repeated measurements (e.g., arterial blood gas parameters) will be analyzed using repeated measures ANOVA or mixed-effects models.

To assess the relationship between respiratory support parameters and clinical outcomes, multivariable regression analyses will be performed, including linear regression for continuous outcomes and logistic regression for binary outcomes.

Time-to-event outcomes will be analyzed using Kaplan–Meier methods and Cox proportional hazards models. A two-sided *p*-value < 0.05 will be considered statistically significant.

Missing data will be handled using appropriate statistical methods depending on the pattern and extent of missingness. Analyses will primarily follow the intention-to-treat principle. Mixed-effects models will be used for repeated measures, as they allow inclusion of incomplete observations. Where appropriate, sensitivity analyses will be performed to assess the robustness of the results.

### 2.8. Duration of the Study

**Figure 6:**
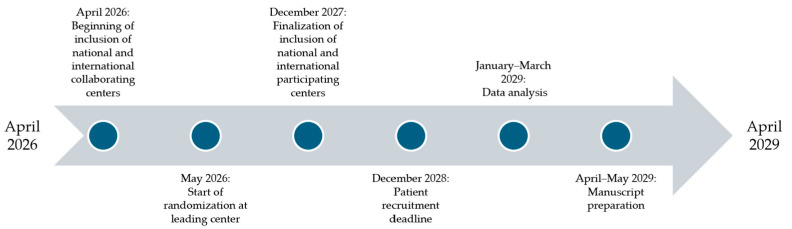
Flowchart depicting the planned duration of the study.

The planned period of the study is from April 2026 to April 2029, with April 2026 being the date of beginning of inclusion of national and international collaborating centers. Randomization at the leading center will begin in May 2026. By December 2027, the inclusion of national and international participating centers will finalize. The patient recruitment deadline is set to December 2028, if the aimed number of participants is not reached prior to the set date. From January to March 2029, data analysis will be conducted. With the completion of data analysis, the manuscript preparation is set for April–May 2029 (Figure 6).

## 3. Results

The results of our study will assess the role of NIV and HFNC as respiratory support strategies during FOB and compare their effectiveness with that of the current standard of care, i.e., COT. In addition, we aim to determine whether, in patients with chronic respiratory failure undergoing FOB, prior stabilization of respiratory parameters improves procedural tolerance, or whether the use of NIV or HFNC may eliminate the need for such preprocedural optimization.

We also aim to identify the optimal NIV and HFNC settings for the studied patient groups, thereby providing a basis for future guidelines on respiratory support during FOB.

Primary endpoint

The primary endpoint is defined as treatment failure, comprising at least one of the following occurring during FOB:

- Need for endotracheal intubation;
- Premature termination of bronchoscopy due to respiratory deterioration;
- Escalation of respiratory support—(COT → HFNC → NIV → IMV).

Secondary endpoints

1. Hypoxemia, decrease in saturation > 5% for more than 30 s: on room air in patients without prior respiratory support, as well as in patients during respiratory support used prior to the procedure;
2. Hypercapnia measured transcutaneous TcCO_2_, increase in TcCO_2_ >10 mm Hg for more than 30 s during the study;
3. New cardiac arrhythmia [>30 s—supraventricular; any ventricular (salvo, bigeminy, etc.)];
4. Hypotension, defined as MAP < 65 or a drop of more than 30% of the baseline value;
5. Complications related to the procedure: glottis damage, pneumothorax, dental trauma, bronchospasm;
6. Need to extend HFNC or NIV therapy after FOB—[time in minutes];
7. Duration of hospitalization, and prolonged hospitalization defined as ≥7 days;
8. Need of ICU admission;
9. Resuscitation;
10. Death.

Study objectives

Verification whether NIV and/or HFNC support during FOB are safer methods of respiratory support in hypercapnic patients than COT.

Finding precise indications based on blood gas analysis for selection of HFNC or NIV in chronic and acute type 2 respiratory failure.

Finding precise settings of HFNC and NIV in patients with type 2 respiratory failure undergoing FOB for diagnostic of therapeutic reasons.

## 4. Discussion

To the best of our knowledge, this study is among the first prospective randomized trials to evaluate respiratory support strategies during FOB specifically in patients with hypercapnic (type 2) respiratory failure.

FOB is routinely used for both diagnostic and therapeutic purposes; however, there is still considerable room to improve procedural tolerance and safety. The limited literature and the lack of clear guidelines regarding the use of advanced respiratory support methods, such as NIV and HFNC, highlight an important area for further investigation.

Adequate oxygenation and hemodynamic stability are key determinants of procedural tolerance. Although these parameters are routinely monitored during bronchoscopy, they do not always provide a complete picture of respiratory status. As reported by Eugen S. Fu et al., inadequate respiratory support may permit the maintenance of apparently acceptable oxygen saturation while acute respiratory acidosis develops as a result of procedure- and/or sedation-related hypoventilation [30]. In addition, sedation may further contribute to CO_2_ retention by reducing respiratory drive and minute ventilation, particularly in patients with pre-existing ventilatory impairment. This raises the question of whether conventional oxygen therapy is sufficient to provide safe and comfortable conditions for FOB while minimizing the risk of escalation to invasive mechanical ventilation.

For this reason, it is important to define optimal respiratory support strategies that enable sedation and FOB to be performed safely and with good tolerance, while minimizing the risk of complications. This is particularly relevant because patients with lung diseases such as COPD or heart failure are especially susceptible to developing significant hypercapnia during sedation [31].

At present, there are no universally accepted disease-specific settings for NIV or HFNC during bronchoscopy in patients with acute respiratory acidosis, chronic respiratory acidosis, or acute exacerbations of chronic respiratory failure. In patients with severe respiratory failure, intubation should be considered before the procedure to allow FOB to be performed safely [19]. In non-intubated patients, however, bronchoscopy may worsen respiratory failure, potentially leading to urgent intubation and ICU admission [29]. In patients with acute respiratory failure, both NIV and HFNC are considerable treatment methods. HFNC provides better comfort and patient perception compare to NIV, according to Pagliaro R. et al. (2024) [32]. Despite good results, the authors suggest further research in establishing optimal HFNC protocols and patient groups, which our study is aiming to do [32].

At the same time, intubation introduces its own technical and clinical challenges. The presence of an endotracheal tube limits the internal airway diameter and may preclude the use of a standard bronchoscope, particularly when tubes smaller than 7.5–8.0 mm are used. Even with larger endotracheal or tracheostomy tubes, insertion of the bronchoscope leaves only a narrow slit-like lumen for ventilation. According to Poiseuille’s law, this markedly increases airflow resistance. As a result, very high ventilation pressures may be required during FOB in intubated patients in order to maintain adequate tidal volume and prevent worsening respiratory failure. These pressures may in turn increase the risk of complications such as barotrauma and hypotension [20]. Furthermore, the use of wider endotracheal tubes to facilitate ventilation may increase the risk of airway trauma, including vocal cord injury. Despite this, the need to maintain ventilation during FOB in intubated patients often necessitates the use of a narrower bronchoscope, which has a smaller working channel and may reduce the effectiveness of therapeutic procedures.

Additionally, the recent literature proves the use of NIV and HFNC as useful methods in prevention of post-extubation respiratory failure. One method does not exclude the other, and their choice should be based on the risk of respiratory failure. A multicenter retrospective study done by Chao W.-C. et al. (2025) showed a significant reduction in 28-day mortality post-extubation mortality with the use of HFNC compared to COT [33,34].

Previous studies of NIV-assisted bronchoscopy have demonstrated improved oxygenation and a reduced risk of hypoxemia compared with conventional oxygen therapy, particularly in patients with hypoxemic respiratory failure [19,35]. However, these studies were generally conducted in relatively small and heterogeneous populations, and focused mainly on hypoxemic rather than hypercapnic respiratory failure [9].

The European Society of Anaesthesiology and the European Society of Intensive Care Medicine currently recommend the use of NIV or HFNC in hypoxemic patients undergoing FOB, as these strategies may reduce the risk of post-procedural intubation compared with conventional oxygen therapy [17]. However, the studies underlying these recommendations were performed in relatively small cohorts and often evaluated specific devices or limited clinical scenarios. This further supports the need for a large multicenter randomized controlled trial in this field. For that reason, we aim to collect intraprocedural data such as SpO_2_, FiO_2_, TcCO_2_, ECG, and heart rate as continuous monitoring, as suggested by Tsai Y.-C. et al. (2025), who stated that hypoxemia as an isolated factor is insufficient to determine HFNC success [36].

Two ongoing randomized controlled trials at our center are currently evaluating HFNC and NIV as respiratory support methods during FOB. The first investigates their use in patients with hypoxemic (type 1) respiratory failure [7]. The second aims to determine whether NIV and/or continuous positive airway pressure (CPAP) improve bronchoalveolar lavage yield in patients with pulmonary diseases such as interstitial lung disease (ILD) and COPD [15].

Although these studies contribute to the growing evidence base on respiratory support during bronchoscopy, they address different clinical scenarios and patient populations from those included in the present study. Patients with hypercapnic (type 2) respiratory failure represent a distinct high-risk group with different pathophysiological mechanisms, including ventilatory failure and CO_2_ retention, which may affect both procedural risk and response to respiratory support strategies.

Therefore, the results of ongoing trials in hypoxemic patients or studies focused on bronchoalveolar lavage performance cannot be directly extrapolated to this population. Rather than duplicating existing research, the present study is specifically designed to address this important gap in evidence regarding optimal periprocedural respiratory support in patients with hypercapnic respiratory failure undergoing bronchoscopy.

In addition, although interest in the use of HFNC and NIV during bronchoscopy has increased in recent years, the available evidence remains limited and heterogeneous, particularly with regard to device settings and their impact on clinically relevant outcomes. Further prospective studies are therefore needed to define optimal management strategies in this setting.

The methodological assumptions of the present study are intended to generate reliable data and to determine whether the proposed diagnostic and therapeutic approaches are safe and clinically beneficial.

Given the clinical importance of this topic, further multicenter collaboration may also be valuable in validating the findings of the present study and improving the generalizability of the results across different clinical settings.

### 4.1. Current State of the Art

Although FOB is a well-established procedure in pulmonology, opportunities remain to improve both procedural conditions and patient tolerance. NIV and HFNC are widely used in the management of respiratory failure and, in some centers, during FOB. However, evidence regarding the optimal settings for these modalities and the patient groups most likely to benefit from their use during FOB remains limited, highlighting the need for further studies in this area.

### 4.2. Impact of Our Protocol on Clinical Practice

HFNC and NIV are being used increasingly in settings where FOB is performed, such as pulmonary departments and intensive care units. These methods may benefit patients by improving gas exchange and reducing the risk of intubation [35,37]. A study such as ours may provide clearer evidence on the most appropriate respiratory support strategy during FOB.

### 4.3. Study Limitations

As a prospective randomized controlled trial, this study has several limitations. Although all enrolled patients have a clinical indication for FOB independent of study participation, the use of different respiratory support modalities during the procedure may introduce additional clinical risk. To minimize this risk, all patients will undergo continuous monitoring, predefined criteria for escalation of respiratory support will be applied, and immediate transition to HFNC, NIV, or IMV will be possible whenever clinically indicated.

Another important limitation concerns the settings of the respiratory support devices, particularly HFNC and NIV. At present, there are no well-established guidelines defining optimal device settings during bronchoscopy, especially in patients with acute or chronic hypercapnic respiratory failure. Therefore, the device parameters used in this study are based on commonly applied clinical ranges and will be titrated according to the patient’s condition. This pragmatic approach reflects real-world practice, but it may also introduce variability between patients and participating centers.

Ongoing studies at our institution evaluating HFNC and NIV in other clinical settings, such as hypoxemic respiratory failure and bronchoalveolar lavage procedures, provided general clinical and methodological background during the development of the present study. However, because their results are not yet available, they were not used to shape the design or assumptions of this trial. The present study should therefore be considered as addressing a distinct clinical question in a specific patient population [7,15].

A further limitation is the possibility of intolerance to the assigned non-invasive respiratory support method, particularly the NIV mask or HFNC interface. In clinical practice, patient discomfort related to these interfaces may occasionally limit the feasibility of the procedure. In our protocol, however, FOB will be performed under intravenous analgosedation, which, in our experience, substantially improves tolerance of both NIV and HFNC and makes such situations uncommon. Nevertheless, any case in which intolerance to the assigned interface prevents completion of the examination will be recorded, classified as a drop-out, and included in the final analysis.

Another potential limitation is the effect of center-specific practice and inter-operator variability on the performance of FOB. Differences in operator experience, procedural technique, and local clinical practice may influence the incidence of procedure-related complications. To reduce this variability, participating centers will follow standardized study protocols for FOB and respiratory support, and all procedures will be performed by experienced bronchoscopists familiar with the study protocol. Even so, some degree of operator-related variability is unavoidable in procedural studies and should be taken into account when interpreting the results.

We also intend to extend the project through multicenter collaboration, which may strengthen the validity and generalizability of the findings and help define the most appropriate respiratory support strategies for this patient population.

Overall, this study is designed to address an important clinical question: whether newer respiratory support modalities can improve the safety, tolerance, and overall conduct of FOB, particularly in patients with more severe respiratory compromise.

## 5. Conclusions

FOB is an important diagnostic and therapeutic tool in pulmonary medicine. Current evidence suggests that respiratory support during the procedure may be further optimized to improve both patient safety and procedural tolerance, reducing the risk of complications while ensuring patient comfort remains an important clinical goal.

We therefore invite leading international centers to collaborate with us in this initiative focused on the management of patients with type 2 respiratory failure undergoing FOB. Centers interested in participating are encouraged to contact the first and corresponding author miko.ryc99@gmail.com, or senior author sz.skoczynski@sum.edu.pl.

## Figures and Tables

**Table 1:** Study group undergoing FOB for therapeutic reasons (Study 1).

Therapeutic FOB (Study 1)			
Group	pH	Randomization—Method of Respiratory Support	*N*—Planned Number of Patients
1	pH < 7.2, but HCO_3_^−^ ≥ 23 mmol/L	IMV or NIV	105
2	7.3 > pH ≥ 7.2, but HCO_3_^−^ ≥ 23 mmol/L	NIV or HFNC	105
3	7.3 ≤ pH < 7.35, but HCO_3_^−^ ≥ 23 mmol/L	NIV, HFNC, or COT	105

**Table 2:** Study group undergoing FOB for diagnostic reasons (Study 2).

Diagnostic FOB (Study 2)			
Randomization 1—Stabilization of ABG Parameters?	ABG Results	Randomization 2—Method of Respiratory Support	*N*—Planned Number of Patients
1 Without stabilization	pCO_2_ > 45 mmHg pH 7.35–7.45 and/or HCO_3_^−^ >27 mmol/L	NIV or HFNC	105
2 Stabilized	pCO_2_ 35–45 mmHg pH 7.35–7.45 and/or HCO_3_^−^ > 27 mmol/L	NIV, HFNC, or COT	105

## Data Availability

The original contributions presented in this study are included in the article. Further inquiries can be directed to the corresponding author.

## Abbreviations

The following abbreviations are used in this manuscript:

FOB	Bronchofiberoscopy
HFNC	High-flow nasal cannula
NIV	Non-invasive ventilation
COT	Conventional oxygen therapy
IMV	Invasive mechanical ventilation
RCT	Randomized clinical trial
ABG	Arterial blood gas
SUM BC	Silesian Medical University Bioethics Committee
GDPR	General data protection regulation
ICU	Intensive care unit
CCS	Chronic coronary syndrome
NYHA	New York Heart Association
PCI	Percutaneous coronary intervention
WHO	World Health Organization
mMRC	Modified Medical Research Council
Apache II	Acute physiology score II
SAPS II	Simplified acute physiology score II
RASS	Richmond agitation-sedation scale
EBUS	Endobronchial endosonography
EPAP	Expiratory positive airway pressure
PS	Pressure support ventilation
COPD	Chronic obstructive pulmonary disease
ILD	Interstitial lung disease
